# Supplementary material for: Resolving the full spectrum of human genome variation using Linked-Reads
Source: Genome Res. 2019 Apr;29(4):635–45. doi: 10.1101/gr.234443.118 (PMC6442396; doi:10.1101/gr.234443.118)
Supplement: Supplemental Material [file supp_gr.234443.118_Supplemental_longranger-2.2.zip › longranger-2.2.tar.bz2!/lr2.2-public/lib/stan/cmdstan/stan/doc/api/html/containers__conversion_8hpp.html]

Stan: src/stan/math/matrix/containers\_conversion.hpp File Reference


|  |  |
| --- | --- |
|  | Stan  2.5.0  probability, sampling & optimization |


- Main Page
- Related Pages
- Namespaces
- Classes
- Files

- File List
- File Members

All Classes Namespaces Files Functions Variables Typedefs Enumerations Enumerator Friends Macros Pages

- src
- stan
- math
- matrix

Namespaces |
Functions

containers\_conversion.hpp File Reference

`#include <stan/math/matrix/Eigen.hpp>`  
`#include <stan/meta/traits.hpp>`  
`#include <vector>`

Go to the source code of this file.

|  |  |
| --- | --- |
| Namespaces | |
|  | stan |
|  | Probability, optimization and sampling library. |
|  | |
|  | stan::math |
|  | Matrices and templated mathematical functions. |
|  | |

|  |  |
| --- | --- |
| Functions | |
| template<typename T , int R, int C> | |
| Matrix< T, Dynamic, Dynamic > | stan::math::to\_matrix (Matrix< T, R, C > matrix) |
|  | |
| template<typename T > | |
| Matrix< T, Dynamic, Dynamic > | stan::math::to\_matrix (const vector< vector< T > > &vec) |
|  | |
| Matrix< double, Dynamic, Dynamic > | stan::math::to\_matrix (const vector< vector< int > > &vec) |
|  | |
| template<typename T , int R, int C> | |
| Matrix< T, Dynamic, 1 > | stan::math::to\_vector (const Matrix< T, R, C > &matrix) |
|  | |
| template<typename T > | |
| Matrix< T, Dynamic, 1 > | stan::math::to\_vector (const vector< T > &vec) |
|  | |
| Matrix< double, Dynamic, 1 > | stan::math::to\_vector (const vector< int > &vec) |
|  | |
| template<typename T , int R, int C> | |
| Matrix< T, 1, Dynamic > | stan::math::to\_row\_vector (const Matrix< T, R, C > &matrix) |
|  | |
| template<typename T > | |
| Matrix< T, 1, Dynamic > | stan::math::to\_row\_vector (const vector< T > &vec) |
|  | |
| Matrix< double, 1, Dynamic > | stan::math::to\_row\_vector (const vector< int > &vec) |
|  | |
| template<typename T > | |
| vector< vector< T > > | stan::math::to\_array\_2d (const Matrix< T, Dynamic, Dynamic > &matrix) |
|  | |
| template<typename T , int R, int C> | |
| vector< T > | stan::math::to\_array\_1d (const Matrix< T, R, C > &matrix) |
|  | |
| template<typename T > | |
| vector< T > | stan::math::to\_array\_1d (const vector< T > &x) |
|  | |
| template<typename T > | |
| vector< typename scalar\_type  < T >::type > | stan::math::to\_array\_1d (const vector< vector< T > > &x) |
|  | |


---

[ Stan Home Page ]

*© 2011–2014,
Stan Development Team.*
